# EXERCISE IN RURAL COMMUNITIES: A QUALITATIVE STUDY OF HOW PAST EXPERIENCES IMPACT CURRENT EXERCISE PRACTICES

**DOI:** 10.1093/geroni/igae098.4179

**Published:** 2024-12-31

**Authors:** Bryant O’Leary, Tasha Shaffer, Brianna Routh, Justin Whitten, Dawn Tarabochia

**Affiliations:** Montana State University, Bozeman, Montana, United States; Montana State University, Bozeman, Montana, United States; Montana State University, Bozeman, Montana, United States; Montana State University, Bozeman, Montana, United States; Montana State University, Bozeman, Montana, United States

## Abstract

As the aging population continually grows, understanding how to keep older adults engaged in exercise is of increasing importance. This is critical as exercise trends show that engagement dramatically decreases with age. Moreover, older adults are more likely to live in rural communities as compared to urban communities (Cohen & Greaney, 2023). Previous studies explored barriers related to the overall decrease in exercise engagement, yet few have focused on older adults’ motivation to maintain exercise practices. The purpose of this project was to explore how past experiences with exercise influence current exercise participation among rural, older adults. Residents engaging in exercise and aged 55+ (n=42) from a western state were recruited using convenience sampling (partnership with local Extension agents and social media outlets). As part of a larger online survey, participants completed an open-ended questionnaire specific to past experiences with exercise. Participant results were analyzed using Van Manen’s (1990) hermeneutical approach with three members of the research team developing themes from participant responses. Trustworthiness was established by a peer reviewer to assess the themes for accuracy. Researchers identified five themes associated with how past experiences influenced current exercise practices. These included health and wellness (18), accessible instruction/resources (17), friends and family (13), community programming (10), and youth activity (4). By understanding past experiences, outreach professionals may find more impactful early or later life supports to promote exercise across the lifespan.

